# Supplementary material for: Modular Clinical Decision Support Networks (MoDN)—Updatable, interpretable, and portable predictions for evolving clinical environments
Source: PLOS Digit Health. 2023 Jul 17;2(7):e0000108. doi: 10.1371/journal.pdig.0000108 (PMC10351690; doi:10.1371/journal.pdig.0000108)
Supplement: S1 Table — (PDF) [file pdig.0000108.s001.pdf]

	number of available values	minimum value	maximum value	description
agem	3192	2.069815	59.794659	Age in months
ex	3192	0.000000	1.000000	Sex
hiv_mom	3185	0.000000	9.000000	Mother's HIV status
ttt_before	1725	0.000000	16.000000	Treatment received before D0 visit
vac_pcv1	1569	0.000000	9.000000	History: 1st pneumococcal vaccine
vac_pcv3	1159	0.000000	9.000000	History: 3rd pneumococcal vaccine
bcx_id	622	1.000000	25.000000	Blood cx ID, D0
pglucose	305	3.100000	14.100000	blood sugar (mmol/L), D0
udip_ket	687	0.000000	4.000000	Urine ketones
udip_nit	686	0.000000	2.000000	Urine nitrites, D0
udip_spec	673	1.000000	6.000000	Urine Spec Grav, D0
urine_cx_id	74	1.000000	15.000000	Urine culture: Microbe identified
urine_type	553	1.000000	3.000000	Urine: type of collection, D0, F23
convulscomplex	3191	0.000000	1.000000	Sign: Complex convulsion ¿=2/24h (d0)
pallor	3192	0.000000	1.000000	Sign: Any sign of anemia (d0)
respdistress	3192	0.000000	1.000000	Sign: respiratory distress (d0)
skin_sev	3192	0.000000	1.000000	Sign: Severe skin or soft tissue infection (d0)
hr1	3142	93.000000	214.000000	Heart rate, initial, D0
hypox	1596	0.000000	1.000000	Vital Sign: Hypoxemia, D0
muaclow	2811	0.000000	1.000000	Vital Sign: MUAC ¡11.5cm & age¿6 months
rr1	3180	20.000000	90.000000	Initial RR entered, D0
temp	3186	37.500000	42.000000	Axillary temperature, D0
waz	3188	-6.950000	5.310000	Weight-for-age z-score
complaint	3192	0.000000	8.000000	NaN
eye	3191	0.000000	1.000000	Symptom: Any eye problem (d0)
abdopain	3191	0.000000	1.000000	Chief Complaint Abdominal Pain, D0
dyspnea	3191	0.000000	1.000000	Chief Complaint Difficulty Breathing, D0
dysuria	3191	0.000000	1.000000	Chief Complaint Dusuria, D0
fev	3192	0.000000	1.000000	Chief Complaint Fever, D0
feveronly	3192	0.000000	1.000000	Chief Complaint Fever Only, D0
loa	3191	0.000000	1.000000	Chief Complaint Loss of Appetite, D0
pharyngitis	3191	0.000000	1.000000	Chief Complaint Mouth/Throat problem, D0
uri	3192	0.000000	1.000000	Chief Complaint URI, DO

S1 Table: Summary statistics of the features of the e-POCT data set.
