# Supplementary material for: Modular Clinical Decision Support Networks (MoDN)—Updatable, interpretable, and portable predictions for evolving clinical environments
Source: PLOS Digit Health. 2023 Jul 17;2(7):e0000108. doi: 10.1371/journal.pdig.0000108 (PMC10351690; doi:10.1371/journal.pdig.0000108)
Supplement: S1 Text — (PDF) [file pdig.0000108.s002.pdf]

Methods

Model architecture and implementation details

The different modules of MoDN are multilayer perceptrons (MLP). MLPs are fully connected feedforward neural networks. The weights and biases of each neuron are optimized via the backpropagation algorithm.

We used *ReLU* activations and one or two hidden layers for each module. The overall architecture thus remained quite simple. For each training data point, the state $\mathbf{S}$ is initialized as a random `PyTorch` tensor of size s. The initial state is also optimized throughout the training process. The input layers are of size $1 + s$, s and s respectively for the encoders, feature decoders and disease decoders. The output layer is of size s for the encoders and 2 for the continuous feature decoders (predicting the mean and standard deviation). The disease decoders and the categorical feature decoders also both have an output of size 2, with a *softmax* activation applied to the output layer to compute the probabilities of the two classes.

Feature decoding

We present here an extended version of the MoDN, including some additional modules to perform 'feature decoding'. This allows the clinician to retrieve the values for previously encoded features or to get predictions for unavailable features. For each feature, we defined a *feature decoder*. Depending on the nature of the feature, they are either *continuous* or *binary*. The feature decoders are applied to the state $\mathbf{S}$ to predict the value of the feature. If feature j is continuous, $\mathbf{F}_j$, $\mathbf{F}_j : \mathbb{R}^s \longrightarrow (\mathbb{R}, \mathbb{R}^+)$. It predicts the mean and standard deviation of feature j. If feature j is binary, we have $\mathbf{F}_j : \mathbb{R}^s \longrightarrow \{0, 1\}$.

In the optimization process of our model we included an auxiliary loss function to train our model to perform feature decoding. The feature decoding loss is made of two components. A 'known' part, corresponding to the features that have already been encoded, and an 'unknown' part, for the features from a later stage in the tree. The 'known' part ensures that the model retains past information, and the 'unknown' infers correlations between encoded features and later features in the tree.

The continuous features are optimized using the *negative log-likelihood* loss and the binary features with the *cross-entropy* loss.

Let $\mathbf{z}_p^t = [(q_p^0, a_p^0), (q_p^1, a_p^1), \ldots, (q_p^t, a_p^t)]$ be the ordered list of (question, answer) pairs for patient p. $c_\theta(\mathbf{z}_p^t, j)$ is the prediction of the model for patient p and feature j given the patient information up to t and a_p^j is the true value of feature j. For the "known" part of the loss, we sum the predicted feature values for features known to the model.

$$feature\_loss\_known = \sum_{p=1}^{N} \sum_{t=1}^{T} \sum_{j<=t} \ell(c_\theta(\mathbf{z}_p^t, j), a_p^j)$$

where ℓ is the negative log-likelihood loss or the cross-entropy loss, depending on the nature of the feature. We sum over all the N patients in the dataset and their corresponding ordered lists $\mathbf{z}_p^t$. Similarly, the unknown part of the model is given by

$$feature\_loss\_unknown = \sum_{p=1}^{N} \sum_{t=1}^{T} \sum_{j>t} \ell(c_\theta(\mathbf{z}_p^t, j), a_p^j),$$

where we sum over the predicted information that has not yet been provided to the model.

As explained in the model optimisation during each SGD step, once all the features in a level of the consultation tree have been encoded, the disease decoders are applied. At this stage, we also apply all the feature decoders and compute the *feature_loss_known* and *feature_loss_unknown*.

Idempotence

We trained the MoDN to be **idempotent**. An operator is idempotent if it has the same effect whether it is applied once or several times. In our setting, it means that the information vector for a patient should not change even if the same feature is encoded twice or more. To enforce that constraint, an additional loss, the *idempotence_loss* was added to the global loss minimized during the optimization. For each feature, we computed the mean squared error between the state after having encoded all the features once and the state after re-encoding the given feature. Let F be the number of different features in the model, $\mathbf{S_p}^b$ the state for patient p once all the features have been encoded once, and $\mathbf{S}_p^f$ the state after re-encoding feature f. Then, the loss is given by

$$idempotence\_loss = \sum_{p=1}^{N} \sum_{f=1}^{F} \left(\mathbf{S}_p^b - \mathbf{S}_p^f\right)^2.$$

Calibration curves

The calibration curves were computed using the observations of the test set. The probability space was split into 13 equally space bins. The $x-$axis shows the mean final predicted probability by the MoDN for the observations in each bin. The $y-$axis shows the proportion of positive diagnoses for the observations in each bin. For a given disease d, let $\hat{P}(d) = p$ be the estimated probability by the MoDN of having d. Then the $y-$axis is an estimate of $P\left(d \mid \hat{P} = p\right)$, the true probability of having d, knowing that the MoDN predicted p.
