# Supplementary figures and images for: Modular Clinical Decision Support Networks (MoDN)—Updatable, interpretable, and portable predictions for evolving clinical environments
Source: PLOS Digit Health. 2023 Jul 17;2(7):e0000108. doi: 10.1371/journal.pdig.0000108 (PMC10351690; doi:10.1371/journal.pdig.0000108)

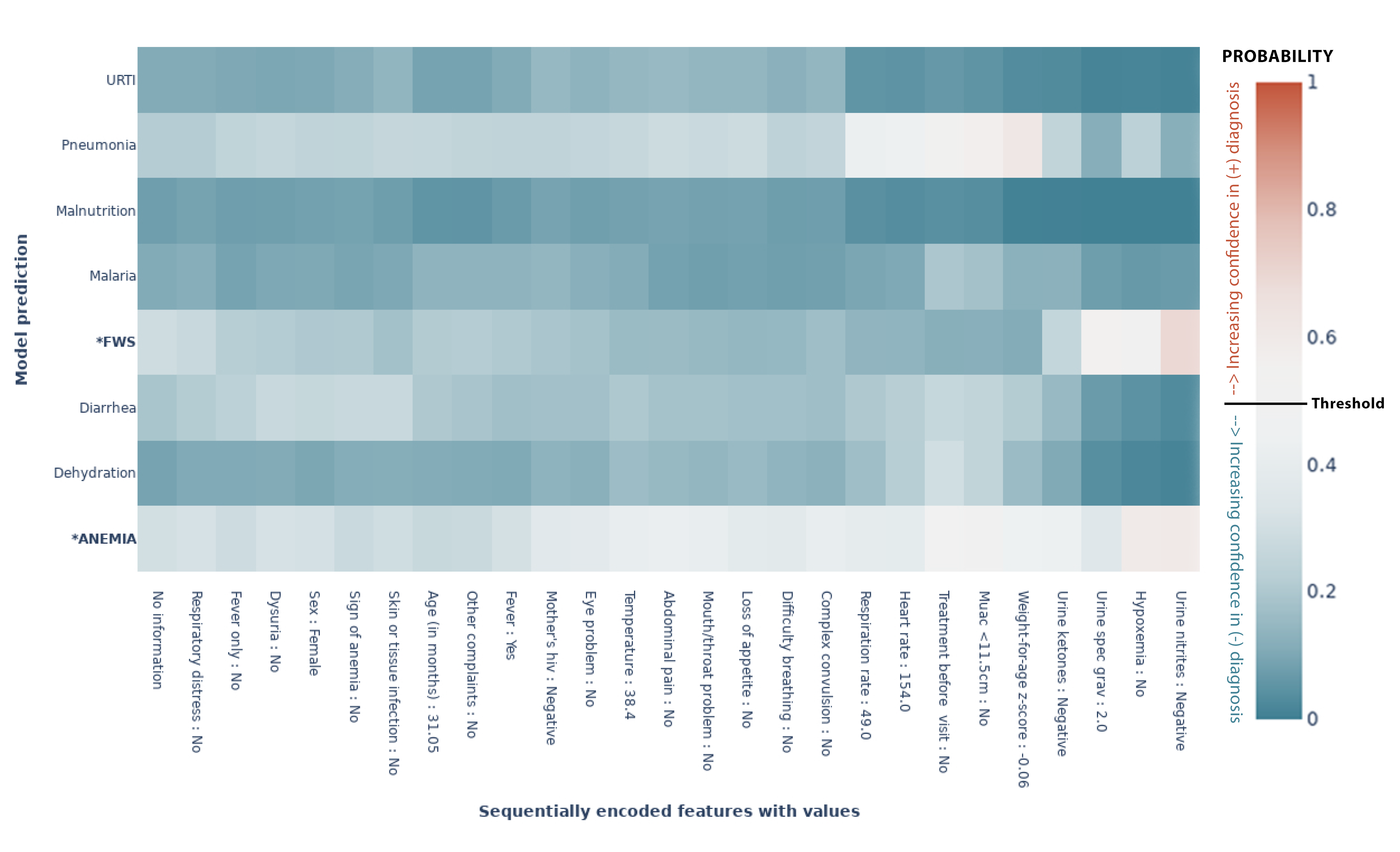

Supplement: S3 Fig — This graph represents a single patient randomly selected from the test set. The y–axis lists the eight possible diagnoses predicted by our model. The true diagnosis of the patient is in bold and marked by an ‘*’. The x–axis is a sequential list of questions asked during the consultation (the response of that specific patient is also listed). In each case the model predicts the true label correctly. The heatmap represents a scale of predictive certainty from red (positive, has diagnosis) to blue (negative, does not have diagnosis), where white is uncertain. This patient has a true diagnosis of FWS and anemia. The model predicts these correctly but with less confidence, as can be interpreted from lighter colours. *: True diagnosis, URTI: Upper Respiratory Tract Infection, FWS: Fever Without Source. (TIFF) [file pdig.0000108.s005.tiff]

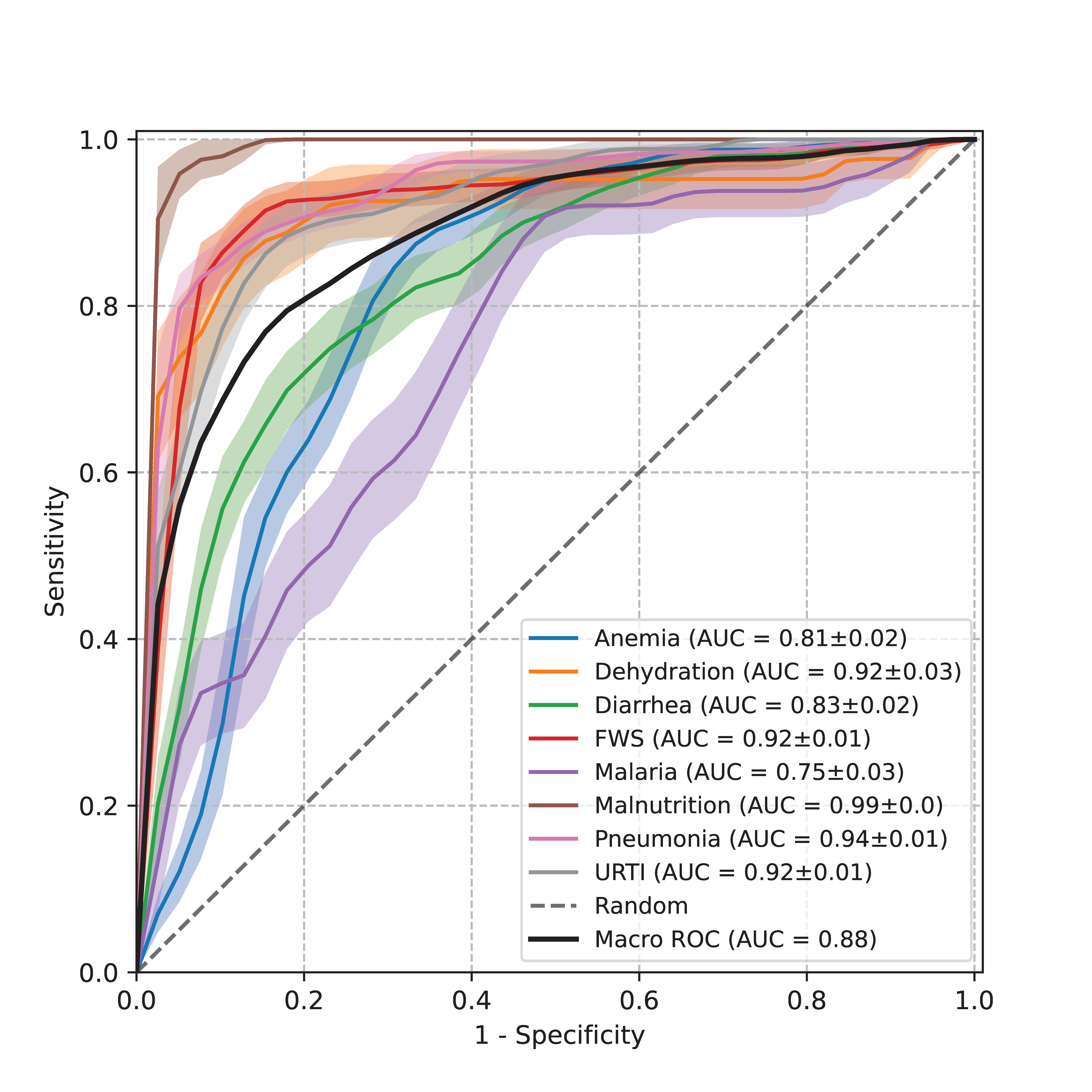

Supplement: S4 Fig — Mean and standard deviation of the AUC and ROC curve for diagnosis prediction computed on the test set, using bootstrapping. (TIFF) [file pdig.0000108.s006.tiff]
