# Supplementary material for: Modular Clinical Decision Support Networks (MoDN)—Updatable, interpretable, and portable predictions for evolving clinical environments
Source: PLOS Digit Health. 2023 Jul 17;2(7):e0000108. doi: 10.1371/journal.pdig.0000108 (PMC10351690; doi:10.1371/journal.pdig.0000108)
Supplement: Attachment — Submitted filename: MoDN-ResponsetoReviewers1_02April23.pdf [file pdig.0000108.s007.pdf]

Point-by-point response

Modular Clinical Decision Support Networks (MoDN)—Updatable, Interpretable, and Portable Predictions for Evolving Clinical Environments

Journal Requirements

1. Please amend your detailed Financial Disclosure statement. This is published with the article. It must therefore be completed in full sentences and contain the exact wording you wish to be published.<br>State the initials, alongside each funding source, of each author to receive each grant.<br>b. If any authors received a salary from any of your funders, please state which authors and which funders.<br><br>If you did not receive any funding for this study, please simply state: "The authors received no specific funding for this work."	Thank you for highlighting this important point.<br><br>We have added the following statement:<br><br>*This work took place within the framework of the DYNAMIC project that is funded by the Fondation Botnar, Switzerland (grant n°6278), MAH received a subgrant for this work.*<br>*The funders had no role in study, analysis, decision to publish, or preparation of the manuscript.*
2. We ask that a manuscript source file is provided at Revision. Please upload your manuscript file as a .doc, .docx, .rtf or .tex.	The .tex file is now included.
3. Please provide separate figure files in .tif or .eps format only and remove any figures embedded in your manuscript file. Please also ensure that all files are under our size limit of 10MB.	All figures have been removed from the manuscript and are now provided in .eps format under 10MB each
4. We have noticed that you have uploaded Supporting Information files, but you have not included a list of legends. Please add a full list of legends for your Supporting Information files after the references list.	Legends for supporting information are now added after the reference list

EPFL iGH intelligent GLOBAL HEALTH

Point-by-point response: Reviewer 1

Does the manuscript meet PLOS DH publication criteria Is the manuscript technically sound, and do the data support the conclusions?<br>---<br>YES	We thank the reviewer for recognizing the novelty, value and methodological soundness of the manuscript.
Has the statistical analysis been performed appropriately and rigorously?<br>--<br>YES	
Have the authors made all data underlying the findings in their manuscript fully available?<br>---<br>YES	
Is the manuscript presented in an intelligible fashion and written in standard English?<br>---<br>YES	

The authors present a development/validation study of a novel CDSS for diagnosis of eight conditions among pediatric outpatients in Tanzania. The CDSS, MoDN, outperforms the authors' chosen baseline models and appears to be relatively robust to "new" (simulated) environments where interoperability may be an issue. The authors explain their modelling technique well, and the use of good figures and illustrative examples benefit the manuscript greatly.	We thank the reviewer for seeing the value and novelty in this work. We are also very happy that the figures and examples are informative.<br><br>We agree with each of the reviewer's subsequent comments and suggestions and have adapted the manuscript accordingly.
In my opinion, the baseline models seem to be set up for failure from the start. For example, the authors discuss the benefits of informative missingness, then choose to perform mean-imputation to generate the training data for the baseline models. Why not encode missingness? Why not use a type of model that generally excels with tabular data and multiclass prediction such as gradient-boosting trees/random forest?	The reviewer highlights excellent points that we had debated ourselves at length.<br><br>The baseline model of a “monolithic” MLP was selected not only because it best represents the individual encoder modules of MoDN but also because it was the best-performing architecture selected from several tested (including those suggested by the reviewer, see below).<br><br>**Why and when imputation was performed?** Monolithic models cannot handle missing values and thus imputation was performed for these baselines. The reviewer rightly identifies imputation as a limitation for the monolithic model: creating an assumed distribution which may bias outcomes. This is one of the reasons why MoDN holds an advantage.<br><br>**Why not encode missingness?** When data is collected in a decision tree, the shape of the missingness may leak the answer to the model as

	it is the shape of the question branch. Thus imputation actually gives our baseline an often overlooked advantage.<br><br>This further highlights the advantage of MoDN, which does not rely on imputation and is not affected by biased missingness, by design.<br><br>**Other model architectures?** The alternate models suggested by the reviewer are excellent suggestions and we indeed tried them as well as others (KNN, Random Forest, etc.). Results were not significantly different from the MLP (with slight differences indicating that MLP and Logistic regression were the best models).<br>To reduce complexity of the text/figure, these other models were not included.<br><br>It is also intended to show the MoDN can act as an architecture that enables interpretability even when using models that are not inherently interpretable via coefficients (such as MLP, which is difficult to interpret as opposed to LR).<br><br>**Multiclass predictions:** Multiclass predictions underperformed as compared to the one-vs-rest approach presented.<br>We thus feel that we have retained the best performing model in the manuscript for the baseline comparison.<br><br>___<br><br>As these are important points, we have now clarified this in the manuscript, adding the following statement:<br><br>*"In addition to MLP and logistic regression, several other baseline architectures were tested, including K-nearest neighbours and random forest. As there was no significant difference, the MLP and Logistic regression were retained for simplicity. This choice also highlights that MoDN can host any model architecture and is able to provide an interpretablity framework despite the deepness of the selected network. This latter point will be particularly advantageous for highly dimensional inputs, such as images."*
Some of the hyperlinks are confusingly labelled, e.g. line 221 refers to "1 and 1" where each "1" hyperlinks to a different section of the manuscript.	Thank you for pointing this out. This has now been corrected.
Lines 250-252/Figure 5: the authors state the points are "close to the line of perfect calibration" - what does "close" mean? It looks quite good, it goes in the right direction, but of the 13 points, 10 are below the diagonal which would suggest the model systematically over-predicts. It would be better to quantify this using e.g. the Brier-score loss or similar. The way binning is performed will also have some impact on how the calibration curve looks, especially for smaller datasets.	The reviewer makes an excellent suggestion which we have adopted.<br><br>We now additionally present the Brier Score loss (Anemia: 0.17, Dehydration: 0.03, Diarrhea: 0.11, FWS: 0.08, Malaria: 0.09, Malnutrition: 0.03, Pneumonia: 0.08, URTI: 0.07 and in average for all diseases 0.08) and have adapted the text to better reflect the tendency for over-prediction as follows:<br><br>*"The calibration curve shows that the model is generally well calibrated, with a tendency to over-predict. In diagnostics this tendency for a higher sensitivity (false positives) is generally more desirable than missed diagnoses (false negatives)".*
Lines 336-345: sequential/continuous updating of beliefs is also a feature of Bayesian Networks: prior beliefs are	Again, we thank the reviewer for the suggestion.<br>We have added the following text to the manuscript to highlight these points.

updated based on conditional probabilistic relationships (e.g. learned model parameters) and the evidence input and allow the impact of each piece of evidence to be quantified. Feature importance for traditional statistical models such as LR is also easily quantified in real time.	*"The feature of continuous updating is comparable to Bayesian Networks but with the advantage of working with any model architecture. Additionally, connecting MoDN modules via the "state" allows the model to carry over any permutation and combination of priors more easily. Specifically, the "belief distributions" are encoded implicitly in the state, without the limitation of this having to be a parametric distribution, and the update rule is learned, rather than computed using Bayes' rule". [...] "Simpler linear models, such as logistic regression are inherently interpretable. MoDN is most advantageous when used as an interpretability framework for deeper models, where inputs are decomposed into single-feature modules and their unique contributions can be explored"*
Tab S1: adjusting the precision of the min and max values for each feature would make the table much more readable. The description of "complaint" is NaN. Various formatting issues with use of D0, DO and d0.	Excellent suggestions.<br>We have cleaned up the table to improve readability.

EPFL iGH intelligent GLOBAL HEALTH

Point-by-point response: Reviewer 2

Does the manuscript meet PLOS DH publication criteria Is the manuscript technically sound, and do the data support the conclusions?<br>---<br>YES	We thank the reviewer for recognizing the novelty and value of the manuscript.<br>The code for the analysis is now publicly available here epfl-iglobalhealth/PLOSDH-MoDN-TrottetVogels2022: MoDN repo for PLOS Digital Health paper (github.com). The anonymized e-poct data can be downloaded from e-POCT | Zenodo.
Has the statistical analysis been performed appropriately and rigorously?<br>--<br>I don't know	
Have the authors made all data underlying the findings in their manuscript fully available?<br>---<br>No	
Is the manuscript presented in an intelligible fashion and written in standard English?<br>---<br>YES	

This is an interesting manuscript presenting an approach how MoDN can achieve to give feedback to the clinician for Clinical Decision Support. As an researcher/clinical pharmacologist I will not comment on the methodological aspects yet focus my comments on the practical aspects including useability of CDSS and meaning of these findings for the clinician.	We thank the reviewer for seeing the value and novelty in this work.<br><br>We find their clinical perspective particularly valuable as a reflection of how such methods would react in real-world settings.
My main comment is that this paper does not – as the title suggests – make clear what this solution means with respect to updating knowledge and interpretation in clinical care. Hence, it is not clear how this work will help the clinician solving actual problems. My suggestion would be to add this information to this manuscript (methods, results and discussion).	We thank the reviewer for highlighting this important point. Indeed this information must be clear in order for readers to understand how MoDN could indeed benefit healthcare workers in practice when using a CDSS.<br><br>We have thus added the following text to the manuscript:<br><br>*"When healthcare workers use static knowledge-based CDSS, the statistical nuances of each question are often blunted into generic binary rules. The inflexible branching logic may also shortcut clinical signs that a clinician finds helpful and oppositely, may force the inclusion of those that they are unable to perform. Indeed poor adherence to CDSS may reflect the user's disagreement with the proposed classification. Our group recently demonstrated this in a CDSS called ePOCT+.*<br><br>*MoDN could allow the user to compose or edit the questionnaire at the bedside, using any combination or number of inputs, while learning their predictive value for the patient through continuous feedback. It would also help nuance the probabilities of differential diagnosis, enabling the user to predict across the branches of the decision tree that would not have been explored in traditional knowledge-based systems."*

	We also edited the abstract to ensure clarity on this critical point.
1. The aim of this study is to present interpretable and predictive feedback to the clinicial. First, please explain how your CDSS works in the routine of the clinicial. At what moment will she/he consult CDSS? Are extra data needed?	We thank the reviewer for this comment, and have added the following text to better describe the CDSS MODN could be integrated within.<br><br>There are many types of CDSS.<br>We have added the following into the text to introduce the concept of a *generalisist CDSS*, which is used throughout the consultation, where the clinician is prompted to collect information that guides them in the diagnosis as well as the treatment and management.<br><br>*These `simple' dichotomous summaries are well adapted to small decision trees with highly specific inputs, but become challenging and inaccurate for more generalist predictions, such as a broad primary assessment of outpatients. Generalist CDSS are intended to be used throughout the full consultation: prompting the probabalistic collection of clinical information and guiding the user to a diagnosis as well as a treatment and management strategy.*<br><br>*The digitalization of CDSS into mobile apps has shown promise in increasing access and adherence to guidelines, while laying the foundation for more systematic data collection but the issues around their prohibitive complexity and limited probabalistic nuance remain. (Roukema2008,Ant2014, Keitel2018, Tan2023 , Pelle2020)*
2. You aim to support the clinician to predict the development of 8 diagnoses: anaemia, dehydration, malaria, diarrhoea, fever, malnutrition, pneumonia, upper respiratory tract infection. Can you please explain how your system is meant to work.	We thank the reviewer for this question, indeed it was not clear, the prediction of the 8 diagnoses were just an example of what MODN can do when integrated within a CDSS. We have clarified this in the manuscript:<br>*A subset of eight diagnoses (targets) and 33 clinical variables (features) were selected in order to ensure interpretable reporting and limit computational cost. The broad range retained is intended to represent the complexity of a generalist CDSS, that collects a large number of clinical variables throughout a consultation and guides the clinician through a consultation to a range of probabalistic diagnoses.*<br><br>And<br><br>*The selected features and targets are examples, and \md can be trained on any number or combinatino of targets and features.*
a. Please elobarate on how you support clinicians. How far before the actual diagnose are they supported. Can your system predict events that will occur in two months (I doubt). Or can they predict malaria once there is a diagnostic test available (in that situation: what is the meaning of CDSS as you already have a diagnosis). It is not clear for me how your system is supposed to work.	Like any predictive model, the predictions of MoDN are constrained to target it was trained on. In the case of the current paper, MoDN is trained on a dataset where the diagnostic targets (8 diagnoses) are made within the scope of an outpatient consultation.<br><br>In in this scope, MoDN will be able to give continuous predictive feedback on all 8 diagnoses after each input (which can be asked in any order or combination) i.e. in the absence of a specific malaria test.
b. What is the predictive value of your system? Sensitivity and specificity. As a clinician I want to be really sure that I will not miss a malaria/pleumonia.	We thank the reviewer for this useful suggestion. We now included the AUC-ROC curves for the prediction of each disease in figure S1.

	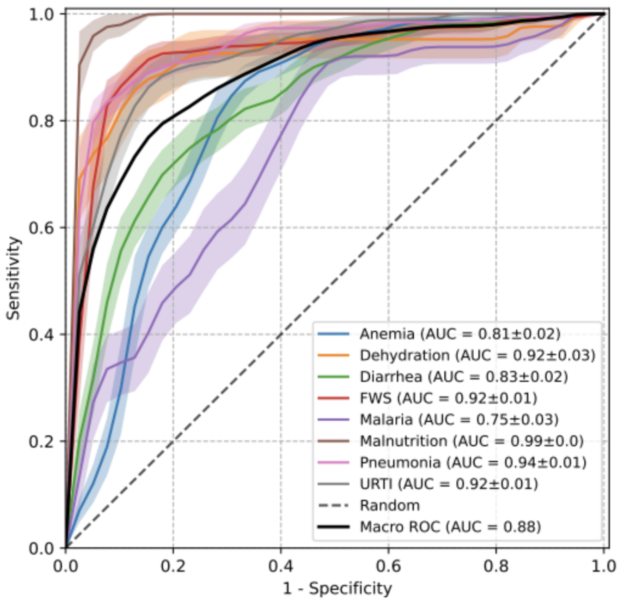
c. What are the requirements with respect to patient data needed. Are there any restrictions with respect to the (patient) data needed? E.g. is the system also accurate as there is no diagnostic test for malaria? Or haemoglobin for anemia?	The reviewer highlights an important advantage of MoDN--there are no requirements.<br>The predictions can be made from any combination or number of inputs and at each point of a consultation. The confidence of the prediction increases as the number of informative inputs increases.<br><br>The final performances listed are given when all questions have been asked.<br><br>The reviewer rightly identifies the inputs of haemoglobin and and malaria RDT as perfect proxies of the response.<br>We could actually incorporate such a perfect proxy into MoDN without trivialising or influencing the predictions made before it is asked. However, for simplicity, these questions have not been included in the question list.<br><br>The question list is provided in annex and they are provided in order
3. Introduction. Some sentences might be somewhat speculative.<br>a. In the introduction (line 11-16) authors make some very general assumptions about CDSS in a way that seems to disqualify lack of available evidence, and use this as motivation for their approach. From a clinical perspective, also expert opinion – for example by elaboration of clinical or pharmacological knowledge (Weersink, BMJ Open; Van Tongeren, Frontiers) – is an important approach to develop the best possible recommendations. The context should further motivate the best approach. In most situations clinicians favor an approach where they can understand why certain	We understand the reviewers' suggestion and have edited both the abstract and introduction of the manuscript to highlight the specific contributions of MoDN and better nuance its contrast to traditional CDSS.<br><br>Additional references are also added to better support the text.

options in clinical decision making are preferred in comparison to others.	
4. Methodology. I am not an expert in this field, but please state that the dataset that is used is big enough for the analysis you used and the data contain enough details to analyse this, and that the outcomes can/cannot be used in other settings. Do you aim to develop general CDS rules for all pediatrics in the world, or only for the local setting?	**Sample size:** The appropriateness of the size of the dataset for a given model can be evaluated in several ways.<br><br>One important indicator is the observed variance of the training loss, calibration curve and confidence intervals over cross validation.<br>As can be seen by our results, the variance in confidence intervals is only a few percentage points, which is acceptable and sufficient to find statistical significance in model comparison.<br><br>**Generalizability:** No CDSS could claim to generalize well to all pediatrics in the world unless it was trained on a data set representing this (unattainable) population. As with any predictive model, the results are only provably valid for the context in which the training set was derived.<br><br>The aim of this manuscript is to introduce a novel and interpretable model architecture that could be used to train models on CDSS-derived datasets.<br>The dataset used serves as an example of the capabilities of this model.
5. Should the model not be tested on a datasource outside the 3192 outpatients in another setting?	External validation is always an excellent suggestion. However, collecting a comparable dataset is a significant effort that is well outside of the scope of this study.<br><br>We are indeed currently collecting an extended dataset using an updated CDSS. This is a 5-year undertaking and we look forward to testing our model on this new cohort!<br><br>In the present manuscript, we have implemented Machine Learning techniques that better ensure the reported results are more representative of expectations on new data. Specifically, using a cross validation technique and a hold-out test set). The hold-out test set was not used for model training and tuning. The results presented in the paper (interoperability, AUROC, calibration, heatmaps,...) were all computed on this test set, thus showcasing model behaviour on unseen data.<br><br>To further ensure good model generalizability, we used 5-times 2-fold Cross Validation during the model tuning phase. Thus after excluding the test data, we split the remaining data randomly into two subsets. Half of this data was used in turn for training and for validation. The model performance is computed on the validation data. By repeating this process (i.e. randomly splitting the data into training and validation subsets), we can use paired t-tests to compare model and baseline performance on unseen data (Dietterich TG. Approximate Statistical Tests for Comparing Supervised Classification Learning Algorithms. Neural Computation. 1998;10(7):1895–1923. doi:10.1162/089976698300017197.).
6. Can you discuss in what situation this model will help pediatricians in their clinical care? What tasks are supported? To what extend and for what diagnoses will this help the patient?	MoDN has various advantages.<br><br>**In what situation will it help clinicians in clinical care?** MoDN creates data-driven predictions on a range of diagnoses that can be updated and viewed after each question is asked.

	Imagine having access ot the heatmap we present in the manuscript during a consultation, where each column becomes available immediately after asking the question to the patient.<br>It allows the clinician to understand the relative predictive value of each question they ask, as well understand the probability of differential diagnoses.<br><br>**What tasks are supported.** We train it on 8 tasks as a example. MoDN has the enormous and unique advantage that it can be trained on any number or combination of features and tasks. I.e. the number of decoders and encoders are not limited.<br><br>**How will this help the patient.**<br>The knowledge of the probabilities of differential diagnoses would allow the clinician to make decisions on asking additional questions to investigate alternative possibilities and thus potentially reduce missed diagnoses.<br><br>The ability to get continuous feedback on the probability of differential diagnoses during a consultation using any number or combination of inputs, allows the clinician the flexibility to CHOOSE the inputs during the consultation. In effect composing the model at the bedside. It also allows them to skip inputs that the patient cannot afford/does not want to answer or that are not available.<br><br>This could greatly optimize the time and resources required to achieve a reasonable confidence in a diagnosis and better nuance the differential diagnoses as well as allow the clinician and patient to better understand the value of their responses and how they contribute to the final diagnosis.
