# Supplementary material for: Modular Clinical Decision Support Networks (MoDN)—Updatable, interpretable, and portable predictions for evolving clinical environments
Source: PLOS Digit Health. 2023 Jul 17;2(7):e0000108. doi: 10.1371/journal.pdig.0000108 (PMC10351690; doi:10.1371/journal.pdig.0000108)
Supplement: Attachment — Submitted filename: MoDN-Response_MinorReview25May.pdf [file pdig.0000108.s008.pdf]

Point-by-point response: Journal Requirements

Please review your reference list to ensure that it is complete and correct. If you have cited papers that have been retracted, please include the rationale for doing so in the manuscript text, or remove these references and replace them with relevant current references. Any changes to the reference list should be mentioned in the rebuttal letter that accompanies your revised manuscript. If you need to cite a retracted article, indicate the article's retracted status in the References list and also include a citation and full reference for the retraction notice.	*The references have now been thoroughly reviewed and corrected*

Point-by-point response: Reviewer 1

Thank you for the opportunity to review the revised manuscript. All my comments have been successfully addressed and I commend the authors on their work.	We are extremely happy to have been able to address the reviewer's concerns and again thank them for the excellent advice.

Point-by-point response: Reviewer 2

First, I would like to thank the authors for answering the questions and the improved version of the manuscript.	We thank the reviewer for seeing the value and novelty in this work.<br><br>We find their clinical perspective particularly valuable as a reflection of how such methods would react in real-world settings.
An issue that has not been solved, and what would improve the clinical applicability of the manuscript is a description how this tool is to be used in the patient journey. Yet is is difficult to understand the added value for clinical practice. In this descriptions the authors should show how this tool is or should be implemented in clinical care, so that it is clear what other steps or actions can be skipped or improved.	The modular design of MoDN makes it agnostic to input, model, and task. This means<br><br>1) **Input agnostic:** A new input (question) can be added and an existing one removed without impacting any modules trained before it. I.e. a module can be skipped at any point and any number or combination of modules can be deployed. This creates enormous flexibility at the bedside.<br>**2) Model agnostic:** we have decided to make all our modules dense networks for simplicity, but they are self-contained and could be various combinations of model architectures supporting various modalities of inputs (images, sound, text etc).<br>**3) Task agnostic:** Eight decoders are tested in this implementation. Each is specific to a diagnostic task. This is not a multi-class model (where all class predictions are predicted in parallel) but

	rather multi-task, where there is a decoder dedicated to the prediction of a single task. The model architectures of these decoders can also be changed and the task can be binary, multiclass, regression, etc.<br><br>With this enormous flexibility in implementation, it is not representative to depict a single implementation.<br><br>We have now added this critical explanation to the text in several prominent places in the manuscript<br>**ABSTRACT:**<br>MoDN is a novel decision tree composed of feature-specific neural network modules that can be combined in any number or combination to can make any number or combination of diagnostic predictions, updatable at each step of a consultation.<br><br>**INTRODUCTION**:<br>The model is flexibly extended during the course of the consultation, adding any number or combination of neural network \textit{modules} specific to each question asked. This results in a dynamic representation of the patient able to predict the probability of various diagnoses at each step of a consultation.<br><br>**METHODS, MoDN:**<br>The algorithm is thus agnostic to the 1) input---accepting any type, number, or combination of inputs, 2) model architecture---where each encoder and decoder can be of any architecture, such as a CNN, MLP, etc., and 3) task---where any number of combination of task-specific decoders can be deployed at any point.<br><br>**CONCLUSION:**<br>This work showcases the various advantages of modularising neural nets into input-specific modules. First composability---where the user can input any number or combination of questions and output any number or combination of predictions.<br>Second, the flexible portability of the modules also provides more granular options to building collaborative models which may address some of the most common issues of data ownership and privacy.<br>Third, modularity also creates an inherent interpretabilty where the sequential deployment of modules provides granular, input-specific interpretable feedback that aligns with the sequential logic of a medical consultation.<br>Finally, MoDN's ability to skip over missing inputs enables it to train on CDSS-derived data irrespective of the presence of biased missingness, and thus for it to be used flexibly across settings with evolving access to resources.

Some other questions I asked have been answered adequately, yet no changes are made in the manuscript. I believe that the manuscript will be improved if these answers will also be included in the manuscript (e.g. questions 2a - how you support clinicians; 2c - requirements with respect to patient data needed; 5. external validation; include as limitation in the text; 6. explain in what situations this model will help.	We thank the reviewer for the opportunity to further clarify our text.<br>We actually did adapt the text accordingly, but the reviewer perhaps could not identify it due to their issue of accessing the track-changes version.<br><br>We are pleased to note that our addition from the above question on the flexibility of use has also addressed questions 2a, 2c and 6.<br><br>Questions 5 (external validation) is now explicitly addressed in the limitations section as follows<br><br>For the IIO experiments, we purposely use an experimental setup that mimics two imperfectly interoperable data sets (i.e. splitting data sets and random feature deletion). We use this instead of an independent IIO dataset to better isolate the effect of the IIO without influence from data-dependent variation in the distribution in each feature across two data sets.<br>"External validation on a larger data set" is desirable in any study. However, we could not find any public patient-level CDSS-derived datasets on a comparable population, even with only a partial overlap of collected features.
I did not receive a track changes version that made it difficult to check the changes in detail.	Track changes was submitted, but perhaps was not accessible via the review platform for technical reasons.
The link provided by the authors with the underlying data could not be opened by me. I could see there was a file posted, yet I was unable to check if this file included the underlying data. I believe the editorial office will further check check this	The link has been tested and is working.
